# Large-Scale Analysis Reveals Gene Signature for Survival Prediction in Primary Glioblastoma

**DOI:** 10.1007/s12035-020-02088-w

**Published:** 2020-09-01

**Authors:** Birbal Prasad, Yongji Tian, Xinzhong Li

**Affiliations:** 1 National Horizons Centre, School of Health and Life Sciences, Teesside University, Darlington, DL1 1HG UK; 2 Beijing Tiantan Hospital, Capital Medical University, Beijing, 100070 People’s Republic of China

**Keywords:** Glioblastoma, Prognosis, Biomarker, Survival analysis, Meta-analysis

## Abstract

Glioblastoma multiforme (GBM) is the most aggressive and common primary central nervous system tumour. Despite extensive therapy, GBM patients usually have poor prognosis with a median survival of 12–15 months. Novel molecular biomarkers that can improve survival prediction and help with treatment strategies are still urgently required. Here we aimed to robustly identify a gene signature panel for improved survival prediction in primary GBM patients. We identified 2166 differentially expressed genes (DEGs) using meta-analysis of microarray datasets comprising of 955 samples (biggest primary GBM cohort for such studies as per our knowledge) and 3368 DEGs from RNA-seq dataset with 165 samples. Based on the 1443 common DEGs, using univariate Cox and least absolute shrinkage and selection operator (LASSO) with multivariate Cox regression, we identified a survival associated 4-gene signature panel including *IGFBP2*, *PTPRN*, *STEAP2* and *SLC39A10* and thereafter established a risk score model that performed well in survival prediction. High-risk group patients had significantly poorer survival as compared with those in the low-risk group (AUC = 0.766 for 1-year prediction). Multivariate analysis demonstrated that predictive value of the 4-gene signature panel was independent of other clinical and pathological features and hence is a potential prognostic biomarker. More importantly, we validated this signature in three independent GBM cohorts to test its generality. In conclusion, our integrated analysis using meta-analysis approach maximizes the use of the available gene expression data and robustly identified a 4-gene panel for predicting survival in primary GBM.

**Electronic supplementary material:**

The online version of this article (10.1007/s12035-020-02088-w) contains supplementary material, which is available to authorized users.

## Introduction

Globally, there were about 330000 incident cases of central nervous system (CNS) cancers with a significant increase in age-standardized incidence rate (17.3%) between 1990 and 2016. However, there was no significant change in age-standardized death rate (2.2%) globally between 1990 and 2016 when about 227,000 deaths were reported due to CNS cancers [1]. In particular, CNS cancer incidence was about 5053 in the UK in 2016 with a 21.6% change in age-standardized incidence rates between 1990 and 2016 [1]. Among these cancers, brain tumour incidence rates in the UK are expected to rise by 6% between 2014 and 2035 [2]. Glioblastoma multiforme (GBM), classified as a grade IV glioma (a brain tumour sub-type) as per the World Health Organization (WHO) classification is the most common and aggressive primary CNS tumour [3, 4]. About 2500 new GBM cases are diagnosed each year in England alone [5]. Currently, standard treatments for GBM include surgical resection followed by radiotherapy and adjuvant chemotherapy [6]. Despite recent advances in treatment strategies, the median survival of GBM patients is still about 12–15 months shorter than most of the other major cancers, e.g. breast cancer [7]. The poor outcome for GBM patients is the worst 5-year overall survival (OS) rate among all human cancers [8, 9].

Over the last decade, an increased focus has been on elucidating the molecular pathogenesis of GBM by identifying its specific molecular signatures and pathways [10, 11]. Some of these molecular genetic alterations, for example, isocitrate dehydrogenase 1 and 2 (*IDH1/2*) mutation and O6-methylguanine-DNA methyltransferase (*MGMT*) promoter methylation, have been recognized as more appropriate diagnostic and prognostic markers, respectively, in GBM than histological appearance alone [11, 12]. However, given the dismal prognosis of GBM, novel molecular signatures that can improve survival prediction and treatment response to better prognostic and therapeutic success are still urgently required.

Recently, large amounts of high-throughput genomic data generated using microarrays and next-generation sequencing (NGS) techniques have been archived on public databases such as Gene Expression Omnibus (GEO, https://www.ncbi.nlm.nih.gov/geo/), ArrayExpress (https://www.ebi.ac.uk/arrayexpress/), The Cancer Genome Atlas (TCGA, https://portal.gdc.cancer.gov) and Chinese Glioma Genome Atlas (CGGA, http://www.cgga.org.cn). These provide us the opportunity and resources to explore, integrate and reanalyse the already existing data for new biomarker discovery and validation. In addition, previous studies have reported a correlation between differentially expressed genes (DEGs), microRNAs, long non-coding RNAs and differentially methylated genes and GBM prognosis and have indicated prognostic value using bioinformatic analysis [13–23], but no consistent model exists. For instance, Zuo et al. (2019) [15] and Cao et al. (2019) [16] identified a panel of 6 and 4 genes, respectively, for prognosis prediction with no genes in common. Multiple studies have also focused on establishing solitary gene-GBM relationship without considering the potential advantage of gene combination which may have limited prognostic and predictive power [13, 14].

To improve prognostic and predictive power, a number of recent studies considered multiple mRNA expression datasets and have identified panels of genes to predict prognosis in GBM patients [15–21]. Despite this, these studies have limited focus on a few datasets. Some studies lacked validation of their panels or models in independent cohorts, whereas there is no proper assessment of sensitivity and specificity of the prognostic models in others. Inclusion of more available datasets as well as application of meta-analysis methods [24, 25] to increase the statistical power of studies as a result of larger sample size can lead to a more robust selection of genes [26]. A robust selection of genes has the potential to improve prognosis prediction and treatment response in GBM. Moreover, these studies are also constrained by small number of normal samples. Furthermore, the majority of GBMs (~90%) develop de novo, i.e. they are primary GBM, and have worse prognosis than secondary GBMs which progress from lower-grade astrocytomas [27]. Hence, considering them separately is important.

In this study, we aimed to robustly identify a gene signature panel for improved survival prediction in primary GBM patients by conducting an integrated analysis on mRNA expression data available on public databases including TCGA, GEO and ArrayExpress. Here, DEGs were discovered from collected microarray datasets by using a novel meta-analysis approach we proposed previously [24], while DEGs from TCGA mRNA sequencing (RNA-seq) dataset were identified by RNA-seq analysis. Based on the common DEGs between microarray and RNA-seq datasets, prognosis-related genes were screened by univariate Cox regression. Among these, by using least absolute shrinkage and selection operator (LASSO) approach with multivariate Cox [28], we identified a survival associated 4-gene signature panel and established a risk score model for survival prediction in primary GBM. Moreover, we assessed the sensitivity and specificity of the model using time-dependent receiver operating characteristic (ROC) curves and validated this signature in three independent primary GBM cohorts.

## Results

### Differential Expression of Genes in GBM

**Fig. 1:**
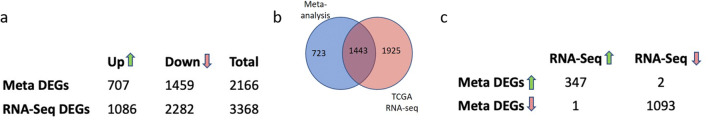
Differentially expressed genes between GBM and normal brain tissues. (**a**) Tabular diagram showing the number of up- and down-regulated DEGs of GBM in meta-analysis for microarray data and TCGA RNA-seq analysis. (**b**) Venn diagram representing the total number of overlapped DEGs between the meta-analysis for microarray data and TCGA RNA-seq analysis. (**c**) Number of up- and down-regulated DEGs in the overlapped DEGs between the two DEGs list (meta-analysis and TCGA RNA-seq analysis)

Meta-analysis identified 2166 DEGs (hereby called as meta-DEGs) in GBM compared with normal brain tissues of which 707 were upregulated and 1459 downregulated. Similarly, 3368 genes were found to be DEGs (hereby called as RNA-seq DEGs) in the TCGA RNA-seq dataset of which 1086 and 2282 were up- and down-regulated, respectively (Fig. 1a). Between meta-DEGs and RNA-seq DEGs, 1443 DEGs (66.62% of meta-DEGs and 42.84% of RNA-seq DEGs) were common (Fig. 1b). Fisher’s exact test (*P*-value <2.2 × 10^−16^) showed that the overlap was statistically significant. All except three overlapped DEGs were regulated in the same direction (up or down) in both approaches suggesting that results were consistent among different techniques (Fig. 1c).

### Prognostic Gene Signature Identification for GBM

By applying univariate Cox regression, we evaluated each common DEG for prognostic significance. Out of 1443 common DEGs (see supplementary file 1, Table S1), 123 were found to be associated with overall survival (OS, Cox *P*-value < 0.05). Thereafter, by using LASSO on these 123 genes, we identified STEAP2 metalloreductase (*STEAP2*), insulin-like growth factor binding protein 2 gene (*IGFBP2*), midkine (*MDK*), protein tyrosine phosphatase receptor type N2 (*PTPRN2*), solute carrier family 43 member 3 (*SLC43A3*), protein tyrosine phosphatase receptor type N (*PTPRN*), SIN-HDAC complex associated factor (*SINHCAF*), methylenetetrahydrofolate dehydrogenase (NADP+dependent) 2, methenyltetrahydrofolate cyclohydrolase (*MTHFD2*), granulin precursor (*GRN*), and solute carrier family 39 member 10 (*SLC39A10*) as the top 10 survival-related genes. The results were similar when LASSO was applied to all those 1443 common DEGs. However, only *IGFBP2*, *PTPRN* and *STEAP2* remained consistently significant (Cox *P*-value < 0.05) for OS after multivariate Cox regression analysis was conducted on the top (5–10) survival-associated genes.

In order to robustly select a panel of genes among these top 10 genes, we fixed the top three *IGFBP2*, *PTPRN* and *STEAP2* and looked for others that significantly improved survival prediction. After multivariate Cox regression was conducted on these combinations, we assessed their performance in survival prediction using the risk score model for each combination and time-dependent ROC curves. Area Under Curve (AUC) at 6 months and 1-year time points was calculated. Finally, a 4-gene signature (*IGFBP2*, *PTPRN*, *STEAP2* and *SLC39A10*) that optimally predicted the OS of GBM patients (Table 1) was identified where *SLC39A10* had negative effect while other three all had positive effects.

**Table 1:** Multivariate Cox regression analysis result for the four genes of the prognostic signature.

Gene	Coeff. (***β***)	HR (95% CI.)	*P*-value
*IGFBP2*	0.323	1.381 (1.189, 1.603)	< 0.001
*PTPRN*	0.226	1.254 (1.096, 1.433)	< 0.001
*STEAP2*	0.288	1.333 (1.095, 1.623)	0.004
*SLC39A10*	−0.385	0.681 (0.488, 0.949)	0.024

### Risk Score Model Based on the 4-Gene Signature Predicts Survival in TCGA GBM Cohort

**Fig. 2:**
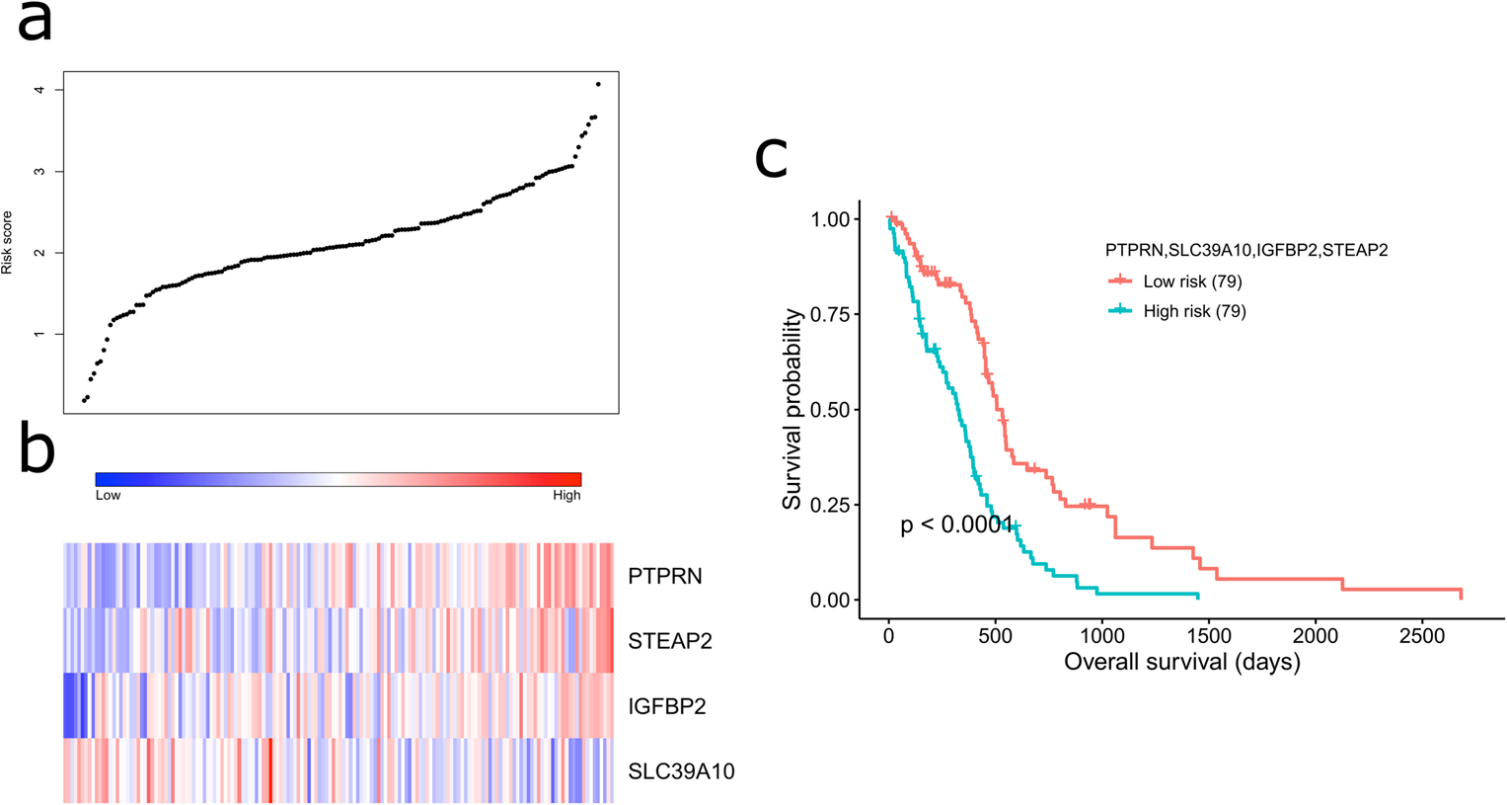
Association between the 4-gene signature and overall survival of GBM patients. (**a**) The distribution of risk scores ordered from low to high. (**b**) Heatmap showing the expression of the four prognostic genes. The expression change from left to right corresponds to the risk score from left to right. (**c**) Survival curves using Kaplan-Meier analysis of overall survival when patients are divided into two risk groups based on median of the risk scores

To assess GBM prognosis based on the 4-gene signature, a risk score model was established to compute risk scores (*r*) for each patient using the following formula (Figure 2a):

$$ r=0.323{e}_{IGFBP2}+0.226{e}_{PTPRN}+0.288{e}_{STEAP2}-0.385{e}_{SLC39A10} $$

where *e*_gene_ is the expression value of a gene in TCGA GBM cohort. Then, based on the median value of risk scores defined by the above formula, the patients in TCGA GBM cohort were divided into a low-risk (79 patients) and high-risk (79 patients) group. In the high-risk group, *IGFBP2*, *PTPRN* and *STEAP2* exhibited a higher expression than in the low-risk group, whereas a lower expression was observed in the high-risk group for *SLC39A10* (Fig. 2b). Kaplan-Meier analysis with log-rank test revealed a shorter survival for patients in the high-risk group than that of the low-risk group (*P*-value < 0.0001, Fig. 2c) suggesting that there might be an adverse association between OS and the risk scores.

**Fig. 3:**
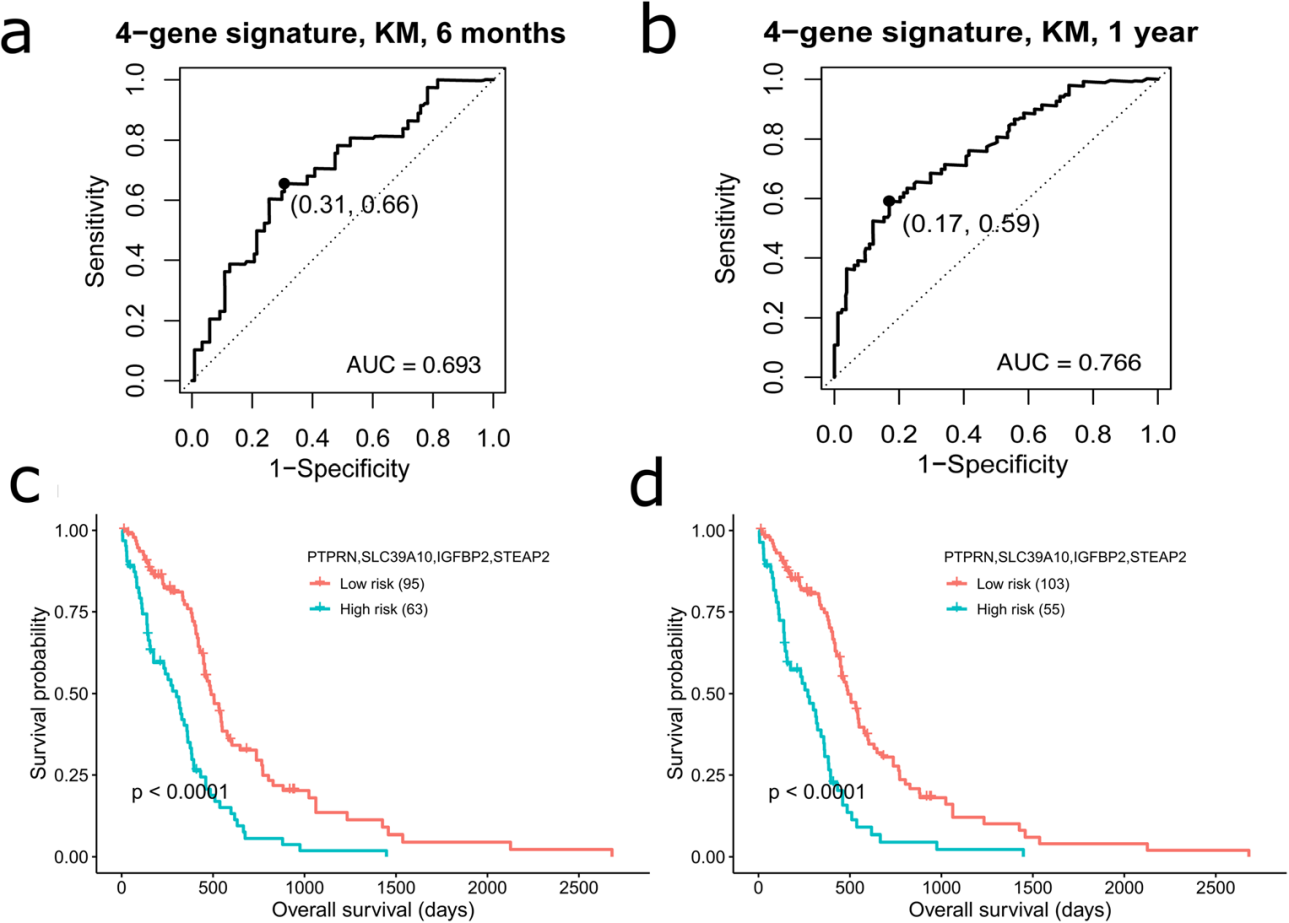
Survival prediction by the 4-gene prognostic signature. (**a**)–(**b**) ROC curves for 6 months and 1 year survival prediction by the four gene signature. Points marked in black represent the optimal cut-off selected for dividing patients into high and low risk groups based on optimization of sensitivity and specificity by maximizing Youden’s index. **(c**)–(**d**) Kaplan-Meier curves of overall survival of the high- and low-risk groups based on optimal cut-off for 6 months (2.27) and 1 year (2.36), respectively

Time-dependent ROC curves showed that the risk scores were capable of predicting survival with high specificity and sensitivity as seen in Fig. 3a and 3b. AUC for the 6 months and 1-year survival prediction were 0.693 and 0.766. The patient’s division into high- and low-risk groups was further improved by using the optimal cut-off selected by maximizing the Youden’s index [29] in the ROC curve which in turn optimizes sensitivity and specificity. For 6 months and 1-year survival prediction, the cut-off was 2.27 and 2.36, respectively. Survival curves constructed using Kaplan-Meier method with log-rank test further suggested a marked difference in OS between the two risk groups (*P*-value < 0.0001) (Fig. 3c and 3d).

### Prognostic Gene Signature and Pathoclinical Factors in TCGA GBM Cohort

Clinical and pathological factors (including age, gender, *IDH* mutation status and *MGMT* methylation status) for which the information was present were studied to assess if the prognostic value of the 4-gene signature was independent of these factors. By using univariate Cox regression analysis, *IDH* mutation status (HR = 0.302, 95%CI: 0.123–0.744, *P*-value = 0.009), *MGMT* methylation status (HR = 0.553, 95%CI: 0.360–0.848, *P*-value = 0.007), and the prognostic signature-based risk score (HR = 2.709, 95%CI: 2.004–3.662, *P*-value < 0.001) were found to be significantly associated with OS whereas age and gender (*P*-value > 0.05) were not (see Table 2). Furthermore, multivariate Cox regression analysis by considering *IDH* mutation status, *MGMT* methylation status and risk score as covariates showed that only risk score (HR = 2.410, 95% CI: 1.569-3.700, *P*-value < 0.001) was significantly associated with patient prognosis (see Table 2b). All these indicated that the 4-gene signature-based risk score was an independent adverse prognostic factor. Overall survival time was considered for both univariate and multivariate Cox regression analysis for all the covariates.

**Table 2:** Pathoclinical factors and the risk score: (a) Univariate and multivariate Cox regression analysis of pathoclinical factors and the risk score for TCGA GBM cohort. *IDH* status, *MGMT* status and risk score were considered for multivariate analysis. (b) *IDH* status, *MGMT* status and risk status at 6 months and 1 year* were considered for multivariate analysis. For each of the features (0) indicates reference subgroup and (1) indicates the other subgroup

Features	Descriptor	No. of patients	Univariate Cox analysis		Multivariate Cox analysis	
			HR (95% CI.)	*P*-value	HR (95% CI.)	*P*-value
(a)						
Age	< 60 (0) ≥ 60 (1)	74 84	1.336 (0.940, 1.899)	0.106	NA	NA
Gender	Female (0) Male (1)	55 103	0.999 (0.691, 1.447)	0.999	NA	NA
*IDH* status	Wild-type (0) Mutant (1)	143 9	0.302 (0.123, 0.744)	0.009	0.526 (0.155, 1.785)	0.303
*MGMT* status	Unmethylated (0) Methylated (1)	67 56	0.553 (0.360, 0.848)	0.007	0.772 (0.488, 1.221)	0.268
Risk score	NA	NA	2.709 (2.004, 3.662)	< 0.001	2.410 (1.569, 3.700)	<0.001
(b).						
*IDH* status	Wild-type (0)	143	0.302 (0.123, 0.744)	0.009	0.245 (0.075, 0.795)	0.019
	Mutant (1)	9			0.250* (0.077, 0.813)	0.021*
*MGMT* status	Unmethylated (0)	67	0.553 (0.360, 0.848)	0.007	0.737 (0.468, 1.159)	0.186
	Methylated (1)	56			0.703* (0.449, 1.102)	0.124*
Risk stat. (6 mon.)	Low (0) High (1)	95 63	2.487 (1.735, 3.566)	< 0.001	2.074 (1.337, 3.217)	0.001
Risk stat. (1-year)	Low (0) High (1)	91 67	2.383 (1.665, 3.411)	< 0.001	1.931 (1.252, 2.978)	0.003

**Fig. 4:**
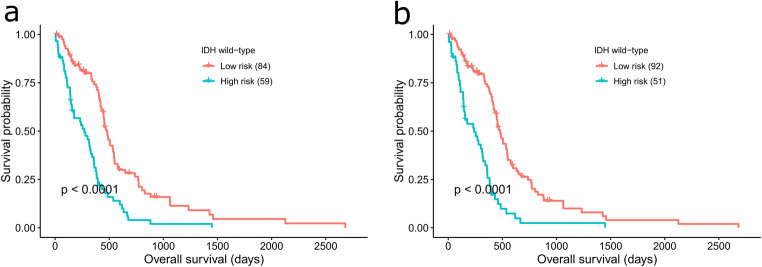
Kaplan-Meier analysis of overall survival of IDH-wt GBM patients in TCGA GBM cohort. (**a**) High- and low-risk groups based on optimal cut-off for 6 months (2.27) and (**b**) 1 year (2.36), respectively

Multivariate analysis with *IDH* mutation status, *MGMT* methylation status and risk status at different time points revealed that *IDH* mutation status and risk status were associated with OS (see Table 2b). Here, risk status refers to the risk group a patient is classified in based on the cut-off. As *IDH* mutation status was identified as OS-associated, the patients were stratified based on this. In the TCGA GBM cohort, since there are very small number of *IDH* mutant patients (9 in total), we only considered *IDH*-wt patients for further stratification analysis to reduce bias. The *IDH*-wt patients were sub-divided into high- and low-risk groups using the optimal cut-off points. Risk score and *IDH*-wt combined survival analysis showed that the *IDH*-wt patients in high-risk group had considerably poor prognosis than the low-risk *IDH*-wt patients (Fig. 4a and 4b).

### Risk Score Model Validation in Independent Primary GBM Cohorts

**Fig. 5:**
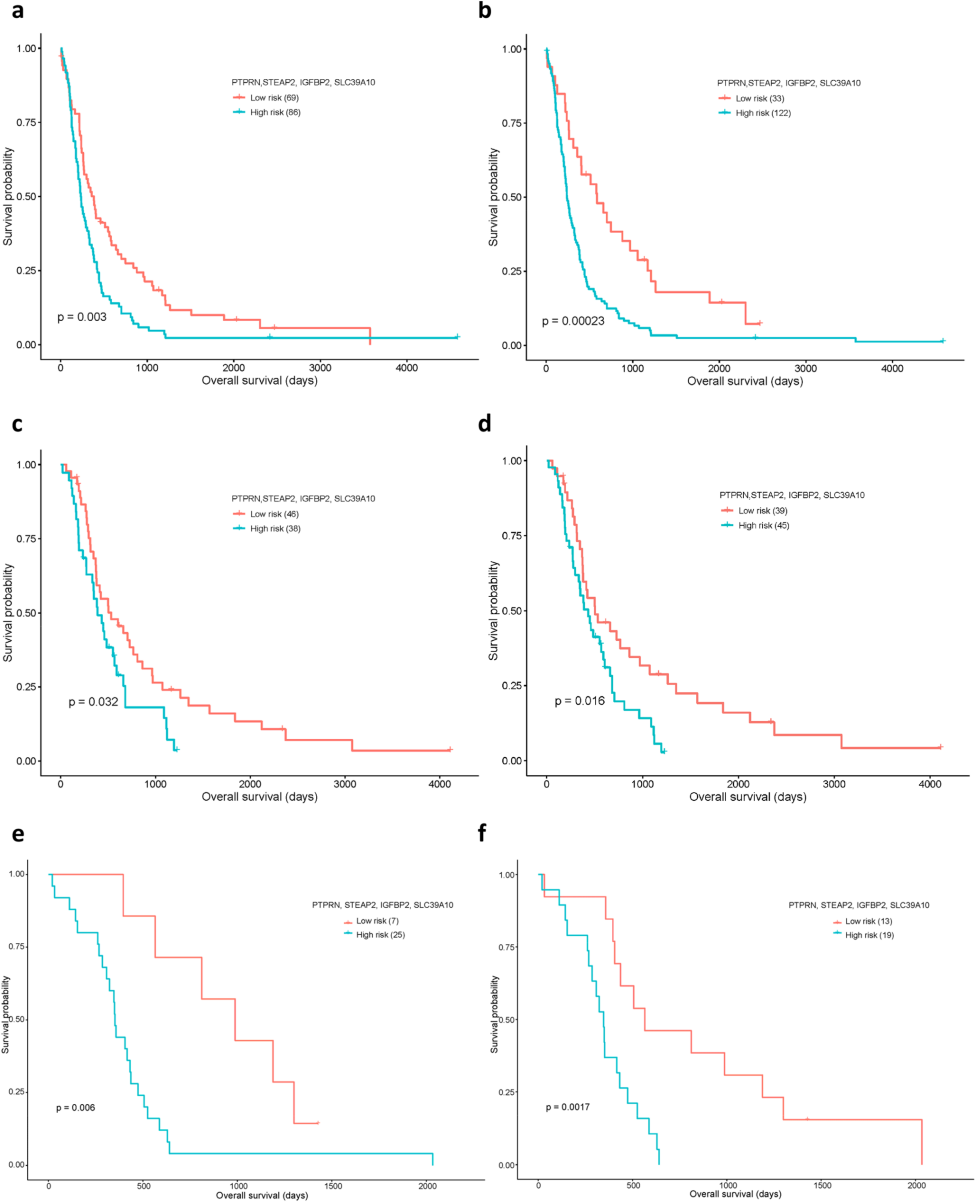
Kaplan-Meier analyses of the four-gene panel in validation datasets. (**a**)–(**b**) For the GSE16011 dataset with optimal cut-off points estimated at 6 months (2.54) and 1 year (3.01) time points, respectively. (**c**)–(**d**) For the CGGA RNA-seq dataset with optimal cut-off points estimated at 6 months (2.42) and 1 year (2.50). (**e**)–(**f**) For the GSE43378 dataset with optimal cut-off points estimated at 6 months (1.33) and 1 year (1.51)

CGGA RNA-seq data including 84 primary GBM samples and two microarray datasets, GSE16011 comprising of 155 primary GBM cases and GSE43378 (32 primary GBM cases) were used for validation of the risk score model. The risk scores were calculated using the same parameters (*β*_*k*_'s) as that of the TCGA GBM cohort. Patients were divided into different risk groups based on the re-estimated cut-off points using time-dependent ROC curve analysis at 6 months and 1 year time points (CGGA: 2.42 and 2.50, GSE16011: 2.54 and 3.01 & GSE43378: 1.33 and 1.51). Performance of the risk scores were then evaluated by Kaplan-Meier analysis. As shown in Fig. 5a–5f, the risk model works well in predicting OS in both GSE16011, GSE43378 and CGGA GBM cohorts and suggested a significantly shorter survival for high-risk group patients compared with that in the low-risk group (*P*-value = 0.00023, 0.0017 and 0.016, respectively, at 1 year).

## Discussion

Several recent reports indicate a potential application of a gene panel-derived risk model in predicting GBM prognosis. Zuo et al. (2019) [15] highlighted the implications of a gene panel as a prognostic predictor in GBM by establishing a six-gene signature risk score model using RNA-seq data from TCGA and CGGA databases but lacked an independent validation of the prognostic signature model. Cao et al. (2019) [16] demonstrated that a 4-gene signature-derived risk score model can predict prognosis and treatment response in GBM patients by conducting a combination analysis on GBM mRNA expression data from two GEO datasets and TCGA, but the sensitivity and specificity of the gene panel in survival prediction were not reported. Yin et al. (2019) [17] identified a 5-gene signature for prognosis prediction in GBM using TCGA RNA-seq cohort and a dataset from GEO database (GSE7696). However, the sensitivity and specificity were assessed using time-independent ROC curves in which the event (disease) status and marker value were considered fixed over time for an individual. In practice, both the disease status and marker value change over time. Moreover, the validation carried out in this study could not be claimed as an independent validation because the TCGA cohort used for validation contained all the samples from TCGA RNA-seq training cohort.

In our study, by integrating multiple gene expression datasets generated by different techniques, i.e. microarray and NGS, and conducting meta-analysis and RNA-seq analysis, we identified four important DEGs, namely, *IGFBP2*, *PTPRN*, *STEAP2* and *SLC39A10* in primary GBM which were also significantly associated to OS. *IGFBP2* was up-regulated and inversely correlated with OS indicating it may act as an oncogene. *SLC39A10* which was down-regulated and positively associated with OS may act as a tumour suppressor gene in GBM. However, *PTPRN* and *STEAP2* were down-regulated and inversely correlated with OS. The risk-score model based on this 4-gene signature performs well in survival prediction for the TCGA GBM cohort and in three independent validation cohorts. Our 4-gene signature-derived risk score model performed better (AUC = 0.766 for 1-year prediction) at classifying the patients into high- and low-risk groups than the 6-gene signature derived and 5-gene signature-derived risk model described in Zuo et al. (2019) [15] (AUC = 0.699 and 0.718 for CGGA and TCGA for 1-year prediction) and Yin et al. (2019) [17] (AUC = 0.708), respectively. It also performed better than the integrated classifier reported in Cheng et al. (2019) [18] (AUC = 0.734 for 1 year).

Of the four genes identified in our prognostic panel, *IGFBP2*, located on the human chromosome 2 (2q35), is a member of the insulin-like growth factor binding protein family and has established roles in GBM. It has been increasingly recognized as a glioma oncogene and a therapeutic target [30, 31]. Overexpression of *IGFBP2* has been found to promote GBM cell migration and invasion and contributes to glioma progression, recurrence, and poor survival in GBM [31]. Holmes et al. (2012) [31] demonstrated that *IGFBP2* expression is closely linked to genes in the integrin and integrin-linked kinase and that these genes are associated with prognosis. Moreover, Liu et al. (2019) [30] established that *IGFBP2* promotes vasculogenic mimicry (VM) formation in glioma cells via regulating *CD144* and *MMP2* expression. VM has been considered as one of the reasons that GBM becomes resistant to anti-VEGF therapy [30]. Our study confirmed significant up-regulation of *IGFBP2* and predicted a poor outcome for patients as shown in previous studies [17, 31–33], thus providing more evidence for further research into its functional roles during GBM progression. *PTPRN* is also located on the human chromosome 2 (2q35) but is interestingly down-regulated in GBM tissue and was associated with poor prognosis as the expression increased. Recent reports have also found that a higher expression of *PTPRN* in GBM tissues is associated with a shorter survival of GBM patients albeit it being down-regulated which was in line with our finding [14, 17, 34]. In oncogenesis, Xu et al. (2016) [35] showed that a high expression of *PTPRN* is associated to tumour growth and proliferation in small cell lung cancer (SCLC). The study further demonstrated that *PTPRN* is a target of the miR-342 and that miR-342 mimics suppressed the expression of *PTPRN* which lead to substantial decrease in SCLC growth. However, the expression level of both miR-342-3p (previously known as miR-342) and *PTPRN* has been reported to be decreased in GBM samples [36, 37], thus warranting future elucidation of other molecular mechanisms involved in *PTPRN* expression and its role in GBM growth and progression. *STEAP2* is located on chromosome 7q21.13 close to *STEAP1* and *STEAP4* genes and plays a role in iron and copper reduction [38]. Its role has been confirmed in prostate and breast cancer in previous studies [39, 40] but has not been studied in GBM. *STEAP2* expression has been found to be significantly increased in prostate cancer, and its knockdown reduced the invasive potential of prostate cancer cells [41]. On the other hand, it is down-regulated in breast cancer tissues, and its low expression was associated with malignant phenotype and poor prognosis [40]. In our study, *STEAP2* was significantly down-regulated in GBM tissues and inversely associated with survival. Moreover, *STEAP2* down-regulation could promote cell proliferation and invasion by activating the PI3K/AKT/mTOR signalling pathway which is also an activated pathway involved in GBM tumorigenesis [40, 42]. This indicates a new research objective for future studies exploring the role of *STEAP2* in GBM growth, progression and prognosis. *SLC39A10* is located on chromosome 2q32.3, and the encoded protein belongs to a subfamily of proteins that show structural characteristics of zinc transporters [43]. High expression levels of *SLC39A10* have been reported to be correlated with invasive behaviour by stimulating cell migration in breast cancer cells [44]. Similar observations have been made in the case of colorectal cancer [45] and renal cell carcinoma [46]. Cao et al. (2019) [16] demonstrated that *SLC39A10* was down-regulated in GBM tissues and positively associated with survival which is consistent with our finding in this study, but its role in GBM progression is poorly understood and requires further exploration in future studies.

A limitation of this study is that in some datasets used for meta-analysis, epilepsy and white matter samples have been deemed as control samples in order to have a considerable number of control samples as compared with the number of tumour samples. The relatively small number of control samples might lead to missing out some potential DEGs. Nevertheless, our meta-analysis will outperform individual microarray studies. Another limitation is that the independent validation is constrained by availability of very few primary GBM datasets that have large sample size (> 50). Nonetheless, our results were validated in the two of the largest independent dataset (to our knowledge) available. For validation datasets, patients were divided into different risk groups based on the re-estimated cut-off. Ideally, the same cut-off for both the discovery and validation datasets should be used, but given the difference in expression values for genes across different datasets generated using different platforms, re-estimation of the cut-off is needed. Furthermore, the role and function of the identified genes in GBM prognosis should be further elucidated in wet-lab experiments.

To summarize, the biological functions and molecular mechanisms in oncogenesis involving the four genes identified have provided hints towards understanding their roles in GBM progression and prognostic and treatment significance of the derived risk score. Moreover, future experimental work is needed to better understand their roles and functions in GBM. The 4-gene panel has promising practical value in the treatment of primary GBM apart from being robust for predicting the survival in primary GBM. In future integrated analysis, we propose to understand the practical value in survival prediction by combining this gene signature with clinical risk factors and other prognostic indices by applying machine learning techniques.

In conclusion, our integrated analysis using meta-analysis approach and two different gene expression techniques maximizes the use of the available gene expression data and robustly identified a 4-gene panel for predicting survival in primary GBM. Multivariate analysis demonstrated that the predictive value of the gene panel-derived risk score was independent of other clinical and pathological features. Hence, the 4-gene panel is a potential prognostic biomarker of primary GBM. Moreover, our findings provide new insights into GBM pathogenesis and prognosis and necessitate future studies.

## Methods

### Gene Expression Data Collection

As for the discovery datasets, public databases GEO (https://www.ncbi.nlm.nih.gov/geo/), TCGA (https://portal.gdc.cancer.gov/) and arrayExpress (https://www.ebi.ac.uk/arrayexpress/) were searched for all primary GBM-related mRNA expression studies of human brain tissue. Studies were selected for analysis if they: (**a**) used clinically diagnosed adult primary GBM patients and (**b**) had at least three control and three tumour samples in their study cohort. Only one biological sample was used for a certain patient in case there were replicates or multiple samples from the same patient. Using our search and selection criteria, we found eight microarray gene expression data sets (GSE4290, GSE12657, GSE13276, GSE19728, GSE90886, GSE108474, GSE116520 and TCGA microarray) with a total of 955 (865 case and 90 control) samples as well as the TCGA RNA-seq dataset with 160 GBM and 5 control samples (Table 3). Seven of the eight microarray datasets were Affymetrix chip generated, whereas one was Illumina chip produced. Corresponding clinical information was also downloaded for these selected studies.

**Table 3:** Information about microarray-generated datasets and number of samples included in our meta-analysis

Datasets	Platform name	Platform ID	Case	Control	Total
GSE2490	Affymetrix Human Genome U133 Plus 2.0 Array	GPL570	81	23*	104
GSE19728	Affymetrix Human Genome U133 Plus 2.0 Array	GPL570	5	4	9
GSE108474	Affymetrix Human Genome U133 Plus 2.0 Array	GPL570	220	28	248
GSE12657	Affymetrix Human U95 Version 2 Array	GPL8300	7	5	12
GSE13276	Affymetrix Human Genome U133A Array	GPL96	5	3**	8
TCGA	Affymetrix Human Genome U133A Array	GPL96	521	10	531
GSE90886	Affymetrix Human Gene Expression Array (Prime View)	GPL15207	9	9*	18
GSE116520	Illumina HumanHT-12 V4.0 expression beadchip	GPL10558	17	8	25

*Epilepsy, **White matter

### Data Pre-processing

For the identified microarray datasets, raw CEL and non-normalized expression files were obtained for the Affymetrix platforms and Illumina HumanHT-12 V4.0 expression beadchip platform generated data, respectively. Each dataset was prepared individually for the meta-analysis starting with removal of outlier samples using box and density plots. Data acquisition and pre-processing were done according to the framework prescribed in Ramasamy et al. (2008) [47]. The datasets were then normalized using the Robust Multi-array Average (RMA) approach [48]. Annotation of the probesets to Entrez Gene IDs and gene symbols was carried out using the manufacturer supplied annotation files. The probesets that did not map to any Entrez Gene ID were removed. Probesets that mapped to multiple genes were removed as well. For genes that matched to more than one probesets, the one with the largest absolute estimated effect size was kept [24]. To remove low expression data noise, a two-step filtering was applied to each dataset. First, a pre-filtering was done using the present/absent call (affy MAS5.0 algorithm) such that the probesets that are present in at least 10% of the samples are kept. For the perfect-match arrays only, probesets with average expression level less than three were discarded. Second, we removed the bottom 5% of average expression values across samples for each dataset. Additionally, for GSE108474 and TCGA microarray dataset, batch-effect correction was applied using the ComBat function in R (sva, version: 3.32.1) as they comprise of a collection of data generated at various centres.

For GBM RNA-seq data, raw counts were downloaded from TCGA database and were annotated by mapping Ensembl IDs to Entrez Gene IDs and gene symbols (org.Hs.eg.db package in R, version 3.8.2). We filtered out the one with no Entrez ID and in the case of multiple matchings, we selected the one with highest aggregated count. Counts per million (CPM) filtering were used to reduce the number of low expressed transcripts [49]. We removed a transcript if five or more samples had less than 0.85 CPM for that transcript. This is analogous to removing any transcripts with less than 40 mapped reads across all samples. The remaining transcripts were then normalized using the trimmed mean of M values (TMM) normalization method and common and tag-wise dispersion were estimated. The edgeR package in R [50] was used for RNA-seq analysis.

### Differential Expression Analysis

We explored DEGs in both microarray and RNA-seq datasets separately.

#### Meta-analysis for Microarray Studies

Differential expression analysis for all the microarray data sets was performed using the novel meta-analysis method described in Li et al. (2015) [24] and implemented as metaUnion package in R (accessed from https://github.com/chingtoe365/metaUnion). Using this method, a combined effect size across studies was computed to identify DEGs assuming normality of the data. We used this approach over the other existing ones [51] because this method accounts for the combined gene sets from all studies included in the meta-analysis. The DEGs between control and tumour samples were selected based on $\sum \limits_{i=1}^n\mid {\log}_2\mathrm{F}{\mathrm{C}}_{\mathrm{i}}\mid /\mathrm{n}>1$ and Bonferroni *P*-value < 0.05 criteria where *n* denotes the number of datasets in which a particular gene was present. However, we only consider a gene as DEG for our final analysis if it was present in at least two datasets included in the meta-analysis to improve the robustness.

#### DEGs from RNA-seq Data

Once the transcripts were normalized and both common and tag-wise dispersion estimated, a negative binomial generalized log-linear model was fitted to the read counts using the glmFit function in R under the edgeR package. DEGs were then selected based on |log_2_FC| > 1 and Bonferroni *P*-value < 0.05 criteria. To obtain the final expression level for each gene, we computed the transcripts per million (TPM) values as log_2_(TPM + 1). A constant factor one was added to account for genes with zero read count in some cases [17].

### Common DEGs and Survival Analysis

**Fig. 6:**
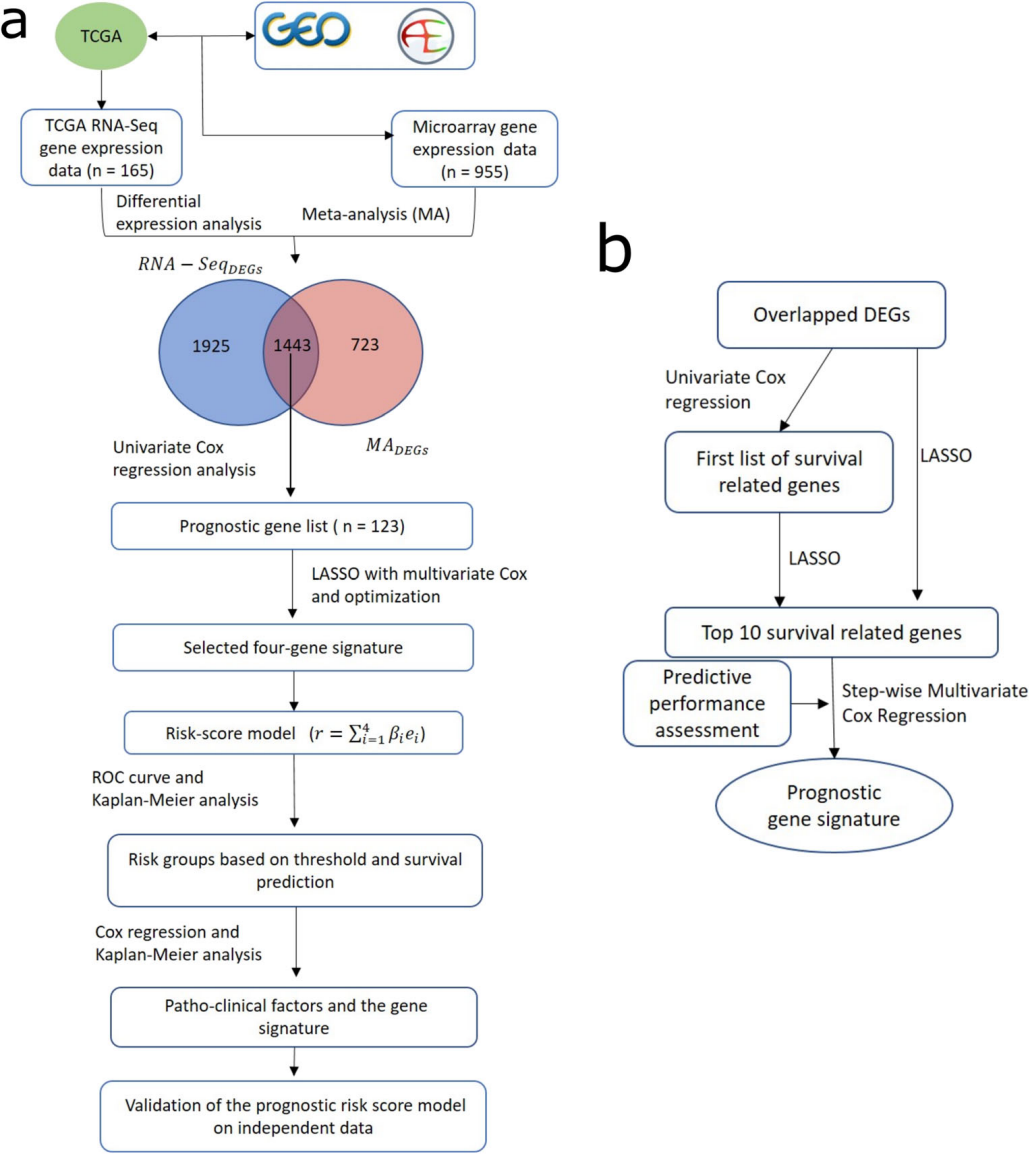
Study workflow. (**a**) Overall steps in the integrated analysis to identify a 4-gene prognostic signature. **(b**) Feature selection step in the workflow. The predictive performance step was carried out for each possible combination of genes from the top 10 survival-related genes

To search for robust DEGs related to GBM, we selected the DEGs that are common in the two DEG lists obtained from microarray meta-analysis and RNA-seq analysis. A two-tailed Fisher’s exact test was used to determine the significance of overlap between these two DEG lists. We also checked for consistency of our results by comparing what percentage of DEGs was regulated in the same direction in the two lists. Next, to evaluate the association of common DEGs with OS, we first conducted univariate Cox proportional hazard regression analysis for each of them in the TCGA GBM (RNA-seq) cohort. The proportional hazard assumption was also checked and found to be appropriate in our case. Second, for the significant genes (Cox *P*-value < 0.05) from the univariate analysis, we used LASSO with multivariate Cox proportional hazards model to robustly and optimally select a panel of genes which were key DEGs associated with OS [28] (Fig. 6b). Pathoclinical features were also assessed for association with the overall survival using the univariate and multivariate Cox regression model. The workflow and schematics of our study are shown in Fig. 6.

### Risk Score Model

Based on the selected survival associated gene signature, we established a risk score (*r*) model [15–17] for prognosis, i.e.

$$ r=\sum \limits_{k=1}^m{\beta}_k\times {e}_k $$

where *m* is the total number of genes in the selected gene signature and *β*_*k*_ and *e*_*k*_ are the multivariate Cox regression coefficient and expression value of the *k*_*th*_ gene in the signature respectively. *r* was computed for each patient using the above formula, and patients were divided into low-risk and high-risk groups. The split into these two risk groups was first based on the median of risk scores and then optimized by choosing the optimal cut-off determined by time-dependent ROC curve analysis using Youden’s index [29]. Kaplan-Meier method with log-rank test was used to analyse survival differences and plot the survival curves for these two risk groups.

### Risk Score Model Validation

For validation purposes, three independent primary GBM gene expression datasets (microarray and RNA-seq) with survival information were downloaded from GEO database (GSE16011, GSE43378) and the CGGA (http://www.cgga.org.cn/), respectively. These datasets were processed by the same workflow as the one used for discovery datasets described above. We used the same *β*_*k*_’s as the one for TCGA GBM cohort for constructing the risk score model for these datasets. Time-dependent ROC curves and Kaplan-Meier method were used to validate the prognostic value of the 4-gene signature for primary GBM patients.

## Electronic supplementary material

Additional file 1: Table S1: Differentially expressed genes identified to be significantly associated to survival in TCGA RNA-seq GBM cohort using univariate Cox regression analysis. (PDF 109 kb)

Additional file 2: Differentially expressed genes identified in our meta-analysis of microarray primary GBM datasets collected from GEO and TCGA databases. (CSV 88 kb)

Additional file 3: Differentially expressed genes identified by RNA-seq analysis in the TCGA RNA-seq GBM dataset. (CSV 251 kb)

## Abbreviations

GBM: Glioblastoma Multiforme
DEGs: Differentially expressed genes
LASSO: Least absolute shrinkage and selection operator
CNS: Central nervous system
UK: United Kingdom
WHO: World Health Organization
IDH1/2: Isocitrate dehydrogenase 1 and 2
MGMT: O^6^-methylguanine-DNA methyltransferase
NGS: Next-generation sequencing
GEO: Gene Expression Omnibus
TCGA: The Cancer Genome Atlas
CGGA: Chinese Glioma Genome Atlas
ROC: Receiver operating characteristic
IGFBP2: Insulin-like growth factor binding protein 2
MDK: Midkine
PTPRN2: Protein tyrosine phosphatase receptor type N2
PTPRN: Protein tyrosine phosphatase receptor type N
SLC43A3: Solute carrier family 43 member 3
STEAP2: STEAP2 metalloreductase
SINHCAF: SIN-HDAC complex-associated factor
MTHFD2: Methylenetetrahydrofolate dehydrogenase (NADP+dependent) 2, methenyltetrahydrofolate cyclohydrolase
GRN: Granulin precursor
SLC39A10: Solute carrier family 39 member 10
OS: Overall survival
AUC: Area Under Curve
CD144: Cadherin 5
MMP2: Matrix Metalloproteinase 2
VM: Vasculogenic mimicry
RMA: Robust multi-array approach
TMM: Trimmed mean of *M* values
