# Supplementary material for: Large-Scale Analysis Reveals Gene Signature for Survival Prediction in Primary Glioblastoma
Source: Mol Neurobiol. 2020 Sep 1;57(12):5235–46. doi: 10.1007/s12035-020-02088-w (PMC7541357; doi:10.1007/s12035-020-02088-w)
Supplement: Supplementary file 1 — Table S1: Differentially expressed genes identified to be significantly associated to survival in TCGA RNA-seq GBM cohort using univariate Cox regression analysis. (PDF 109 kb) [file 12035_2020_2088_MOESM1_ESM.pdf]

Large scale analysis reveals gene signature for survival prediction in primary glioblastoma

Birbal Prasad[1], Yongji Tian[2] and Xinzhong Li[1*]
[1]National Horizons Centre, School of Health and Life Sciences, Teesside University, Darlington, DL1 1HG, UK. Email: B.Prasad@tees.ac.uk; X.Li@tees.ac.uk
[2]Beijing Tiantan Hospital, Capital Medical University, Beijing, 100070, P.R. China. Email: tyjpumc@163.com
*Correspondence: Email: X.Li@tees.ac.uk, Phone: +44-01642738451

Table S1: Differentially expressed genes identified to be significantly associated to overall survival in TCGA RNA-seq primary GBM cohort using univariate Cox regression analysis. Proportional Hazards (PH).

Entrez id	Symbol	Coeff.	P-value (Cox)	Pvalue (PH)	Cox score
5799	PTPRN2	0.45769272	0.00016514	0.31875768	14.3608838
5798	PTPRN	0.23051635	0.00022437	0.28436601	13.7789477
29015	SLC43A3	0.51903681	0.00026632	0.52829078	13.5783937
261729	STEAP2	0.30419255	0.00055515	0.8214233	12.109393
3706	ITPKA	0.38460537	0.00053507	0.85395322	11.9256858
10769	PLK2	0.32511119	0.00090101	0.58467762	11.011755
9601	PDIA4	0.44730502	0.00096047	0.24450891	10.8644232
55240	STEAP3	0.24662399	0.00108345	0.48061731	10.768553
4192	MDK	0.33032632	0.00121318	0.72814847	10.4298297
9645	MICAL2	0.30685979	0.00139258	0.68104526	10.2084063
84557	MAP1LC3A	0.40067483	0.00171189	0.52861755	9.93126611
27122	DKK3	0.36385562	0.00180981	0.56385622	9.76825228
79605	PGBD5	0.34079112	0.00202848	0.59146914	9.51124832
10797	MTHFD2	-0.3774165	0.00202081	0.46644883	9.43567818
11014	KDELR2	0.53284095	0.00202691	0.13156959	9.38511768
2896	GRN	0.46351579	0.00241801	0.40077134	9.16626864
58516	SINHCAF	-0.2943193	0.00342146	0.37256291	8.62159549
3485	IGFBP2	0.18438201	0.00384212	0.52078182	8.45500719
2990	GUSB	0.42493186	0.00396909	0.78485689	8.32233912
66004	LYNX1	0.28358166	0.00413864	0.2788438	8.2233959
23007	PLCH1	0.31013741	0.00521866	0.44436013	7.87626581
81031	SLC2A10	0.27970861	0.00541799	0.29006077	7.84615986
6938	TCF12	-0.3121634	0.00542033	0.09431318	7.75515793
4745	NELL1	0.21232528	0.00604489	0.91631501	7.65867213
143888	POGLUT3	0.30746599	0.00637759	0.18520193	7.47574223
5999	RGS4	0.16830399	0.0068341	0.99730495	7.34184986
9956	HS3ST2	0.18414994	0.00727977	0.48060938	7.25966823
5034	P4HB	0.42476092	0.00772312	0.92209902	7.07072079
4478	MSN	0.29421018	0.00841107	0.4549524	6.99769879
26240	FAM50B	0.25321864	0.00857862	0.76639805	6.97853976
219738	FAM241B	0.25518884	0.00835011	0.09278555	6.96027825
6804	STX1A	0.2555039	0.00877121	0.96344354	6.852664

80381	CD276	0.38663391	0.00915067	0.81648296	6.79138202
202559	KHDRBS2	-0.2997335	0.01107306	0.99916708	6.70041247
84282	RNF135	0.38939468	0.01084585	0.75388348	6.49201641
5058	PAK1	0.36872538	0.0112301	0.35374711	6.40355783
54472	TOLLIP	0.49701246	0.01183416	0.84295018	6.33231973
302	ANXA2	0.21099156	0.01287264	0.17940874	6.22382455
51148	CERCAM	0.32297366	0.01250339	0.05254425	6.22095766
23096	IQSEC2	0.30706989	0.01279024	0.40052628	6.20918008
9369	NRXN3	0.22570358	0.0130655	0.80364912	6.19675546
5954	RCN1	0.37124776	0.01379168	0.29707222	6.07118974
2014	EMP3	0.19011688	0.01462608	0.657461	6.04559508
23601	CLEC5A	0.17870939	0.0150832	0.30899859	5.93406131
221424	LRRC73	0.27498325	0.01468791	0.30027118	5.90973568
79057	PRRG3	0.24308966	0.01591542	0.158364	5.88119729
25798	BRI3	0.54008938	0.01554205	0.20645667	5.84723868
9737	GPRASP1	0.23635813	0.01578771	0.39825386	5.8274776
57181	SLC39A10	-0.4249265	0.01657431	0.11245005	5.70389889
5782	PTPN12	0.41269414	0.01686011	0.08253254	5.69309832
57535	KIAA1324	0.27842097	0.01847005	0.81146019	5.58120434
158931	TCEAL6	0.14958175	0.01859074	0.85502318	5.58017937
8534	CHST1	0.21011452	0.0185762	0.22301047	5.54657891
2335	FN1	0.18767955	0.01872407	0.62617105	5.52936656
8407	TAGLN2	0.21181065	0.01999721	0.83925362	5.46419196
4313	MMP2	0.19934009	0.01954602	0.618111	5.46373078
10627	MYL12A	0.31334288	0.02016709	0.84080312	5.4139299
112609	MRAP2	0.1615869	0.02040285	0.29024964	5.39894447
56666	PANX2	0.2522143	0.02056659	0.48373764	5.36182212
100506311	HOTAIRM1	0.14077947	0.02155084	0.20464281	5.31307058
400745	SH2D5	0.23047712	0.02147096	0.75336884	5.30750782
158038	LINGO2	0.3506936	0.02235499	0.80915713	5.3002967
3223	HOXC6	0.18576427	0.02193853	0.24337413	5.28529598
6447	SCG5	0.26594183	0.02148004	0.20311541	5.22364382
84918	LRP11	0.3233582	0.02257705	0.13671302	5.20676735
55466	DNAJA4	0.28480672	0.02253491	0.25778402	5.19810073
26470	SEZ6L2	0.15312953	0.02391251	0.43719258	5.12969094
8714	ABCC3	0.12393905	0.02377074	0.12005697	5.12723601
7079	TIMP4	-0.1503839	0.02400273	0.68394053	5.09956382
30061	SLC40A1	-0.2362434	0.02469517	0.61214006	5.0460199
142940	TRUB1	-0.3962722	0.02623315	0.48217419	4.93728199
121601	ANO4	0.21503642	0.0268528	0.39928826	4.92920651
23122	CLASP2	-0.2589087	0.02689852	0.64949459	4.90613135
1475	CSTA	0.1800176	0.02734029	0.6238541	4.89031603
6275	S100A4	0.15988816	0.02704278	0.42663358	4.88830811

7277	TUBA4A	0.18676438	0.02753934	0.22754671	4.86238675
5577	PRKAR2B	0.21779111	0.02775198	0.10257045	4.85524843
79772	MCTP1	0.27967757	0.02786587	0.52599694	4.82804691
51330	TNFRSF12A	0.18328068	0.02821612	0.51482773	4.8219489
57095	PITHD1	0.53719972	0.02860417	0.16363531	4.80969615
26049	FAM169A	-0.2631391	0.02919892	0.09956723	4.76092607
11332	ACOT7	0.27029858	0.02971167	0.59388248	4.73183152
977	CD151	0.24343262	0.03095754	0.39096314	4.67940186
9674	KIAA0040	0.18747901	0.03090234	0.43779996	4.66237784
871	SERPINH1	0.21372673	0.03154193	0.27509411	4.59983931
4828	NMB	-0.1556896	0.03229636	0.26362447	4.59448194
376267	RAB15	0.24974554	0.03253551	0.55082613	4.57999411
54675	CRLS1	-0.4410457	0.03346914	0.32937045	4.53970039
195814	SDR16C5	0.36345873	0.03415684	0.49141567	4.53476005
7045	TGFBI	0.14176659	0.03335856	0.60416707	4.50612067
783	CACNB2	0.21889763	0.03406593	0.90188261	4.49913719
3213	HOXB3	0.12104934	0.03509082	0.8310725	4.46818162
57451	TENM2	0.1628748	0.03532277	0.71309796	4.46727262
128434	VSTM2L	0.12882247	0.03517498	0.09904473	4.45758538
10630	PDPN	0.1403172	0.03582549	0.85276611	4.4024386
22924	MAPRE3	0.33511753	0.03590508	0.96108037	4.38854251
814	CAMK4	0.25421051	0.03687624	0.18850836	4.35094025
56341	PRMT8	0.26180407	0.03784767	0.24563647	4.33298134
2185	PTK2B	0.22936735	0.03769598	0.81305992	4.31030049
57628	DPP10	-0.1453427	0.03919809	0.49761501	4.28797137
4318	MMP9	0.10371612	0.03879877	0.93105544	4.28527375
9922	IQSEC1	0.25302527	0.03862211	0.51462114	4.28119699
83857	TMTC1	0.25013919	0.03955209	0.47509474	4.23968056
5355	PLP2	0.17144537	0.03963507	0.83510101	4.2243656
7205	TRIP6	0.22711431	0.04086105	0.75767434	4.21820589
23154	NCDN	0.21413767	0.04160179	0.8644598	4.12730236
6142	RPL18A	-0.2227203	0.04330387	0.56853763	4.10248649
9750	RIPOR2	0.20848524	0.04344265	0.30029558	4.09332064
57574	Mar-04	0.17734965	0.04407092	0.61287911	4.07210509
55332	DRAM1	0.23556418	0.04471646	0.34827487	4.05238481
8871	SYNJ2	0.18846823	0.04446732	0.68197923	4.05197494
8324	FZD7	0.15437289	0.04476755	0.47130151	4.04457652
118429	ANTXR2	0.25285991	0.04438733	0.1873521	4.03868121
2051	EPHB6	0.14600222	0.04480247	0.62618333	4.03614141
55653	BCAS4	0.38891023	0.04479445	0.46771734	4.03002857
5351	PLOD1	0.24615539	0.04504525	0.77874007	4.02243935
25818	KLK5	0.41046103	0.04734203	0.62604299	4.01327959
55603	TENT5A	0.20553525	0.04564648	0.94414432	3.97900897

80212	CCDC92	0.4116828	0.04653768	0.45473277	3.95090659
399668	SMIM10L2A	0.21065363	0.04849151	0.61858737	3.89990432
2039	DMTN	0.17196923	0.04867008	0.61719247	3.88373089
23504	RIMBP2	0.19140243	0.04967572	0.829056	3.86603285
283638	CEP170B	0.19658635	0.04980987	0.6567976	3.83675836
